# Beta-Caryophyllene Enhances the Anti-Tumor Activity of Cisplatin in Lung Cancer Cell Lines through Regulating Cell Cycle and Apoptosis Signaling Molecules

**DOI:** 10.3390/molecules27238354

**Published:** 2022-11-30

**Authors:** Emad A. Ahmed, Hamad Abu Zahra, Rebai Ben Ammar, Maged Elsayed Mohamed, Hairul-Islam M. Ibrahim

**Affiliations:** 1 Biological Sciences Department, College of Science, King Faisal University, Al-Ahsa 31982, Saudi Arabia; 2 Laboratory of Molecular Physiology, Zoology Department, Faculty of Science, Assiut University, Assiut 71515, Egypt; 3 Laboratory of Aromatic and Medicinal Plants, Center of Biotechnology of Borj-Cedria, Technopole of Borj-Cedria PBOX 901, Hammam-Lif 2050, Tunisia; 4 Department of Pharmaceutical Sciences, College of Clinical Pharmacy, King Faisal University, Al-Ahsa 31982, Saudi Arabia; 5 Pondicherry Centre for Biological Science and Educational Trust, Pondicherry 605004, India

**Keywords:** Beta-Caryophyllene (BCP), cisplatin, synergistic chemotherapy, lung cancer cell lines, A549

## Abstract

Beta-Caryophyllene (BCP), a natural bicyclic sesquiterpenes, is an abundant biomolecule in red pepper and other plants. Recently, it was reported to reduce the growth and the proliferation as well as enhance the apoptosis in numerous cancer cells, including colorectal, ovarian, bladder cancer and lung cancer. On the other hand, the combination therapy of cisplatin (CDDP) with other phytochemical compounds has synergistically enhanced the killing effect of CDDP on several types of cancer. In the current model, we have tested the role of BCP in enhancing the anti-tumor activity of CDDP on lung cancer cell lines. The results showed that BCP is not toxic at moderate doses and it can prevent lung cancer progression in doses above 75 µM. However, when being combined with CDDP, BCP improved the former chemotherapeutic function through regulating cell cycle, apoptosis and EMT signaling molecules. Gene and protein expression analysis showed that the combined treatment of CDDP and BCP significantly upregulated the level of the cyclin-dependent kinase inhibitor, CDKN1A, and the inhibitor of the apoptosis, BCL-xl2. In addition, the combination treatment reduced the protein level of the apoptosis regulator, BCL-2. Moreover, BCP appears to prohibit the EMT process that is associated with CDDP chemotherapy since the combination treatment induced a significant increase in the level of the epithelial cell marker E-cad that was reduced in CDDP-treated cells. In agreement with that, the combined treatment managed to modulate the effect of CDDP on the mesenchymal transcription factor ZEB-2. Additionally, molecular docking has been conducted to check the virtual interaction of BCP with these and other signaling molecules, but only cyclin-dependent kinase CDK6 was found to virtually bind with BCP, and at four sites with higher and stable biding energy (−7.8). Together, these data indicate that BCP enhances CDDP chemotherapeutic function through regulating the cell cycle, the apoptosis and EMT signaling molecules.

## 1. Introduction

Beta-Caryophyllene (BCP) is a naturally occurring sesquiterpene can be extracted from several plants, including red pepper, rosemary, hops, and numerous essential oils and cannabis. Recently, it has been reported to reduce the growth and the proliferation rate of numerous cancer cells, including colorectal cancer [1], ovarian cancer [2], human bladder cancer [3] and lung cancer [4,5,6]. Beta-Caryophyllene exerting anti-proliferative activities in lung cancer cells A549 and NCI-H358 cells has been attributed to the induction of G 1 phase cell cycle arrest through downregulating cyclin D1, and other cyclin-dependent kinases, which was associated with upregulating p21CIP1 and p27KIP1 [5]. In another study, BCP of chili pepper was found to inhibit non-small cell lung cancer (NSCLC) growth, and to promote their apoptotic rate and increase the level of apoptotic markers (cleaved caspase-3 and BAX) and anti-oxidant factors (SOD, CAT and GPx) [4]. However, the chemical composition analysis of Bulnesia sarmientoi plant extract (BSE) and the subsequent bioassays using A549 and H661 lung cancer cells indicated that BCP and α-guaiene, (−)-guaiol were responsible for the anti-cancer activity of BSE. In addition, mechanistic assays have shown that BSE displayed strong inhibitory effects on the migration and invasion of A549 and H661 cancer cells [6].

Cis-dichlorodiammineplatinum [II] (cisplatin) is one of the most routinely used chemotherapeutic drugs that are effective in the treating several types of tumors, including lung cancer, esophageal cancer, gastric cancer and colorectal cancer [7,8]. However, even at moderate doses, CDDP is not only cytotoxic to cancer cells but also to normal cells, which is a problem associated with extensive usage of CDDP [9,10]. In addition, CDDP is more poorly tolerated than other chemotherapeutic drugs and many tumors were shown to develop resistance against its chemotherapeutic activity [11]. On the other hand, the combined treatment of CDDP with other biomolecules has improved the outcomes in cancer patients and potentiated the therapeutic effect of CDDP towards tumor cells that enabled the usage of higher doses of CDDP and reduce the toxic side effects in various malignancies [12,13,14].

Lung cancer is a worldwide leading cause of death. There are two major subtypes of lung cancer, small-cell lung cancer (SCLC) and non-small-cell lung cancer (NSCLC); the latter is approximately 80–85% of lung cancer cases. Various methods, such as chemotherapy, surgery, and radiation therapy, have been used to treat lung cancer patients. However, more efficient therapeutics and strategies still needed to be developed for treating cancer.

In the current study, the role of BCP on sensitizing the anti-tumor activity of CDDP in lung cancer cell lines. Beta-Caryophyllene was found to reduce the effective anti-cancer dose of CDDP and improved CDDP chemotherapeutic function through regulating the cell cycle kinases, the apoptosis and EMT signaling pathways.

## 2. Results

### 2.1. Cytotoxicity of Beta-Caryophyllene and Cisplatin and Their Synergistic Effect

**Figure 1:**
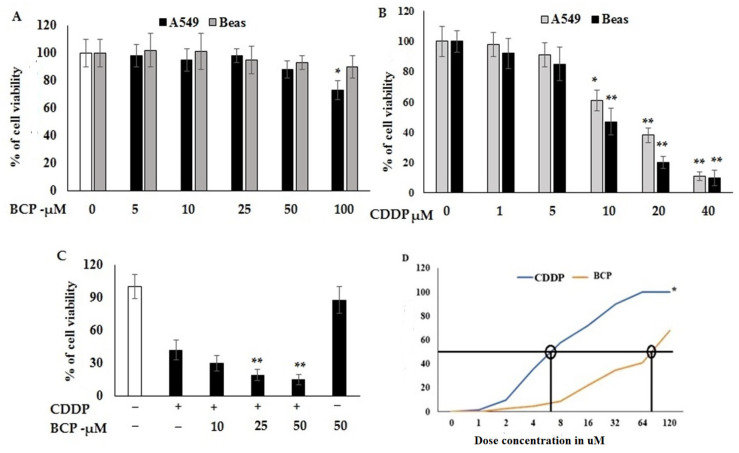
Effect of Beta-Caryophyllene alone and in combination with cisplatin on proliferation activity of lung cancer cell line, A549, and normal lung cell line, Beas. After 48 h of treatment, the control group (DMSO) on the extreme left is assigned 100% viability and cells proliferation was performed using MTT assays. Viability of cells treated with BCP (**A**) and CDDP (**B**). Cells were treated with various concentrations of BCP in the presence or absence of CDDP (**C**). Synergistic dose based on the IC_50_ values was confirmed for both BCP/CDDP at 75 µM and 7 µM, respectively (**D**). Values shown are mean ± SD for three independent experiments. * *p* < 0.05, significant variation and ** *p* < 0.01 high significance variation versus DMSO- or CDDP-treated cell lines.

Cytotoxic properties of BCP and CDDP each separated or both in combination on A549 and Beas cell lines were studied using an MTT-based method (Figure 1). Data indicated that BCP is not significantly toxic to A549 and BEAS cell lines in doses below 100 μM. However, A549 was significantly sensitive (*p* < 0.06) at BCP concentration 100 μM, which indicated that BCP is not toxic to lung cancer cells at lower doses (Figure 1A). On the other hand, the cytotoxic properties of CDDP on BEAS and A549 cell lines showed a significant effect at 46% (±3.2) on BEAS and 44% (±4.3) against A549 cell lines (Figure 1B). Evaluating the combined cytotoxic effects of BCP with CDDP on lung cancer cell line and normal lung cell line showed significantly higher cytotoxic effect (Figure 1C). The dose of CDDP showed potential cytotoxicity at IC_50_ 12 μM, this dose was reduced to be around two-fold lower when combined with 50 μM of BCP in a synergistic manner (Figure 1D). These results point out that BCP could improve the cytotoxic effect of CDDP and then solve a problem associated with CDDP treatment, since it is toxic at high doses to both cancer cells and normal cells.

### 2.2. Effect of Beta-Caryophyllene and Cisplatin on Migration and Invasion Activities of Lung Cancer Cell Lines

**Figure 2:**
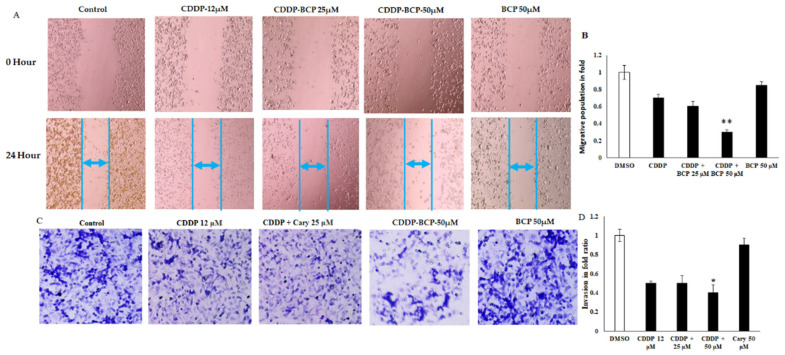
Beta-Caryophyllene reduces tumorigenic properties of A549 lung cancer cells. CDDP and BCP (12 and 25 μM) alone or in combination evaded the migration and invasion of A549 lung cancer cells (**A**,**B**). The migrated cells population from different treated cell lines were quantified at a fixed area. (**C**) The invaded cells were stained with Giemsa and counted in four different microscopic fields (**D**). Invaded cells were quantified using manual counting. Values shown are mean ± SD for three independent experiments. * *p* < 0.05 compared to control vs. BCP alone. ** *p* < 0.01 significant difference. The invaded cells were calculated as the following; 50 cells = 1 fold, as in control and the treated cells value was 75 cells, so it was calculated as 1.5 fold. Values shown are mean ± SD for three independent experiments. * *p* < 0.05 and ** *p* < 0.01 significance and high significant differences, respectively, relative to CDDP-treated cells.

Apart from the cytotoxicity, angiogenic characteristics of tumor microenvironment, including cancer cells migration and invasion, play significant role in cancer progression and recurrence. Migration and invasion are integral process associated with tumor metastasis; in this study, scratch wound distance was significantly lower in untreated control and significantly higher in CDDP-treated cells at dose of 12 μM. Interestingly, the combined therapeutic treatment of BCP and CDDP inhibited the cells migration in the scratch wound site and enhanced a significant increase in the distance between the two edges of scratch compared to A549 cell lines treated with CDDP or BCP (Figure 2A,B). In the meantime, matrigel drop invasion assay showed effective anti-invasive properties of combined treatment that induce a decrease in the invasion activity of A549 cell lines (Figure 2C,D). These factors were further validated by the potential migration marker BCL2, using quantitative real-time PCR.

### 2.3. Effect of Combination Treatment on Mitochondrial Membrane Potential of A549 Cell Lines

**Figure 3:**
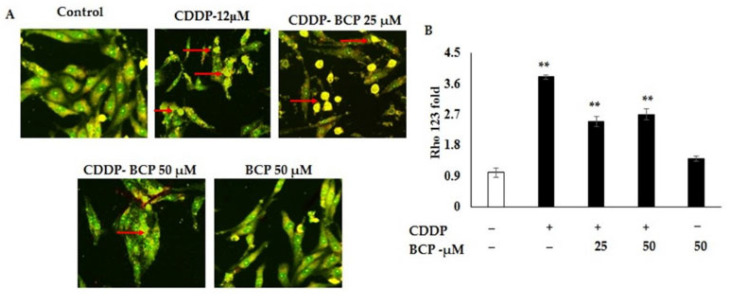
BCP abrogates metabolic activity of A549 lung cancer cells. Rhodamine 123 staining explores the mitochondrial membrane potential of cancer cells. (**A**,**B**) A549 cells were grown on a cover slip-pasted 12-well plate and exposed to CDDP and BCP (12 and 25 μM) alone or in combination and treated for 12 h. (**A**) Cells were incubated with methanol fixed Rho-123 and quantified the excited illumination using fluorescent microscope (Leica). (**B**) The mitochondrial damage and the membrane integrity are shown; less damage and high integrity (green color), more release of content and less integrity (yellow color, arrows), 20× magnification. Values shown are mean ± SD for three independent experiments. ** *p* < 0.01 high significant differences, respectively, relative to DMSO-treated cell lines.

The activation of apoptotic markers such cell cycle regulators and mitochondrial damage are the key points of anti-cancer therapy. Loss of mitochondrial transmembrane potential is a crucial step in the intrinsic apoptosis pathway. In this study, the effect of BCP and CDDP alone and in combination was evaluated on the mitochondrial membrane potential loss in A549 lung cancer cell lines using Rh0-123 fluorescent dye. The results of BCP and CDDP alone and in combination on mitochondrial membrane potential are shown in Figure 3A,B. As seen in fluorescent emission, the combined treatment of CDDP and BCP induced more release of the mitochondrial contents and induced a significant decrease in membrane integrity (Figure 3B). Then, combination treatment actively enhanced the membrane damage and increased the release of mitochondrial content in A549 cell lines.

### 2.4. Beta-Caryophyllene Interacts with the Inhibitor of Cyclin Dependent Kinase-1A, CDKN6

**Figure 4:**
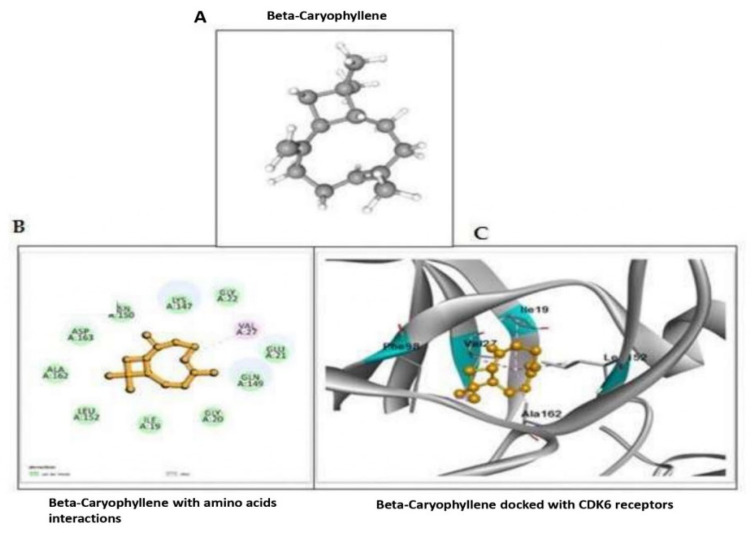
Beta-Caryophyllene interacts with the inhibitor of Cyclin Dependent Kinase-1A, CDKN6 CDK6 with a stable binding energy around –7.8 at four possible binding sites. (**A**) 3D structure of Beta-Caryophyllene CID: 5281515. (**B**,**C**) in silico interaction of BCP with CDK6 PDB ID: 4EZ5 and it shows effective binding and was calculated by the formation of bonds and docked amino acid residues.

To that end, BCP improves the chemotherapeutic effect of CDDP in lung cell lines but still more information about the involved mechanistic action was needed. Therefore, we have conducted several molecular docking designs including checking the possible interaction between BCP and other signaling molecules involve in cancer progression of inhibition. Our results showed negative binding possibility between BCP and these molecules, including E-Cad, ZEB2, BCL-2 and BCL-xl. In further analysis, we have seen a robust and strong binding between CDKN6 and BCP, with a stable binding energy around –7.8 at four possible binding sites (Figure 4 and Table 1). The molecular docking data have indicated that BCP could inhibit the CDKN-1A through binding to CDK6 and then enhance the chemotherapeutic effect of CDDP.

### 2.5. Beta Caryophyllene Enhances Cisplatin Effect on Cell Cycle, Apoptosis and EMT Signaling Molecules

**Figure 5:**
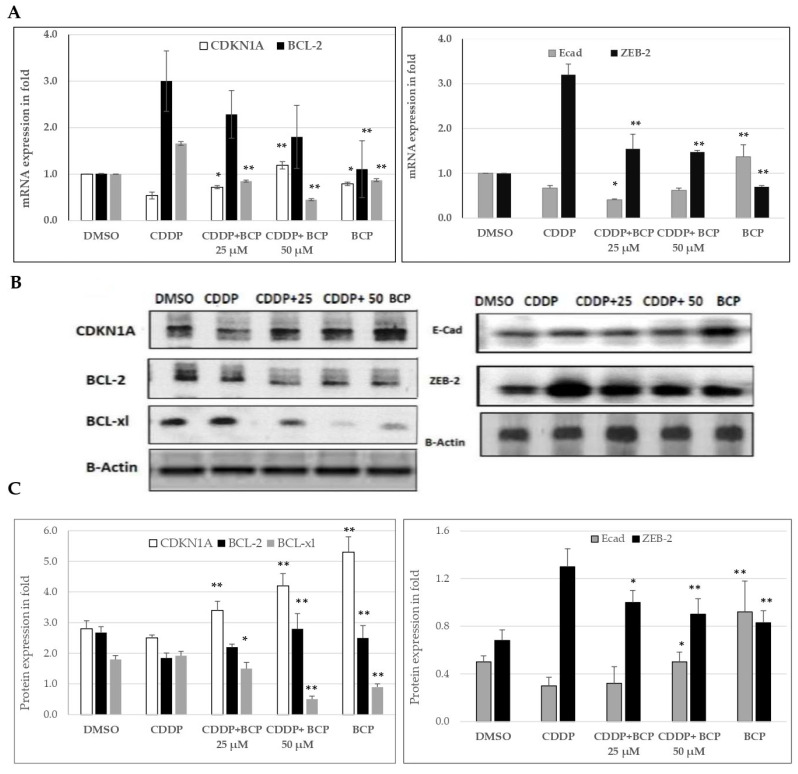
CDDP and BCP regulate the apoptotic and the cell cycle mediators of A549 lung cancer cell lines. (**A**) mRNA expression of CDNK1A, E-Cad, ZEB2, BCL-2 and BCL-xl was quantified using quantitative real-time PCR and GAPDH was used as internal control. The difference in mRNA expression between treated groups is shown in folds. (**B**,**C**) Immunoblots of CDNK1A, E-Cad, ZEB2, BCL-2 and BCL-xl protein were estimated and β actin used as internal control. The difference in protein level between treated groups is shown in folds. Data are expressed as mean ± SD and the differences between variables were analyzed using student *t*-test. The difference was considered to be significant *, when *p* < 0.05 and highly significant **, when *p* < 0.01, versus CDDP-treated cell lines.

To further confirm the effect of BCP on the expression of cell cycle, apoptosis and EMT signaling molecules, mRNA expression of CDNK1A, BCL-2, BCL-xl, E-Cad and ZEB2 was quantified using quantitative real time PCR and GAPDH was used as internal control (Figure 5A). In the meantime, protein expression of these molecules was estimated using Western blot analysis (Figure 5B,C). Interestingly, in lung cell lines and at the gene and the protein expression level, the inhibitor of cell cycle, CDKN1A, was found to be activated by combined treatment of CDDP (12 µM) and BCP (25 and 50 µM, Figure 5) relative to the CDDP-treated cells. In parallel with that, the combined treatment downregulated the protein level of the apoptosis regulator, BCL-2, and upregulated that of the inhibitor of the apoptosis, BCL-xl2. However, the gene expression levels showed similar expression to the protein levels for BCL-xl.

On the other hand, the combined treatment of BCP with CDDP induced a significant increase in the level of the epithelial cell marker E-cad that was reduced in CDDP-treated cells, and thus, BCP appears to prohibit the EMT process that is associated with CDDP chemotherapy. Consistent with that, the combined treatment significantly reduced the level of transcription factor, ZEB2, which was elevated after CDDP treatment. ZEB-2 is a promotor of cancer progression and EMT and then combined treatment managed to modulate the effect of CDDP on the mesenchymal transcription factor ZEB-2. Together, these data indicate that BCP enhances CDDP chemotherapeutic function through regulating the cell cycle, the apoptosis and EMT signaling molecules. 

## 3. Materials and Methods

### 3.1. Cell Lines Culture, Cell Proliferation and MTT Assay

The human lung cancer cell line A549 and Beas normal lung cell line 5000 cell/well in 96-well plates were cultured under standard conditions (5% CO_2_, 37 °C, 95% humidity) in DMEM medium supplemented with antibiotics, as indicated in our previous work [15]. Beta-Caryophyllene was dissolved in DMSO and added to medium depending on the required concentration. Images of these cell line are shown in Supplementary Materials. After 24 h of plating, cells were treated with various concentrations of BCP and CDDP, each alone or both in combination. After 48 h of treatment, cell proliferation assay was done using MTT assays. The MTT reagent (20 μL) was added to the treated cell lines and incubated for 4 h, the media were then removed and results were scored at 490 nm using a microplate reader. The effect of BCP on cell viability was evaluated as the proportion of viable BCP-treated cells relative to the viable vehicle-treated control cells and those arbitrarily deemed to be 100%.

### 3.2. Cell Migration Assay (Wound Healing Assay)

Cancer cells migration was studied using the recovery of the scratched wound in mm. Cell lines were seeded in the culture medium (5 × 10^4^ cells/well) in 24-well plates. Cells were grown to 80% confluence, rinsed with phosphate-buffered saline (PBS) and then starved for 6 h in serum-free medium. The wounds were created using a sterile 100-µL pipette tip. Subsequently, all wells were washed with media to remove cell debris. The cells were then treated with the indicated dose. Then, images were captured at the inverted microscope (magnification: ×200), and the migration activity of cells in a fixed wounded area was calculated with Image J software (ImageJ, Bethesda, MD, USA).

### 3.3. Cell Invasion Assay (Transwell Assay)

Invasiveness of cancer cells was studied using a Boyden chamber insert well model. Cell invasion was assessed using transwell cell culture Boyden chambers, according to the manufacturer’s protocol. The experiment was conducted using 12-well gelatin cell culture inserts (BD Biosciences) with a polyethylene terephthalate membrane (8-µm porosity) coated with Matrigel Basement Membrane Matrix (100 µg/cm^2^). The inserts were incubated for 6 h at 37 °C, and before each assay, 100 µL of cells (5 × 10^4^) were seeded in the upper chamber in serum-free media treated with BCP, CDDP or both at the indicated concentrations. Then, 700 µL of DMEM medium supplemented with 10% FBS was added to the lower chamber. Cells were then incubated for 24 h at 37 °C, the attached cells to the top surface of the membrane were removed using a cotton swab, and the cells on the bottom of the membrane were fixed in cold methanol (75%) for 15 min and washed with PBS three times. After that, cells were stained with Giemsa (30%) stain solution and washed with PBS. Then, cells in five randomly selected fields were counted under a light microscope at 20× objective magnification. The number of migrated cells was tallied by optical microscopy (magnification: 200×) and manual counting, cells were scored in folds (50 cell = 1 fold). All assays were performed in triplicate.

### 3.4. In Silico Analysis

The potential interactions of BCP with human CDKN1A and CDK6 and other signaling molecules was examined using docking analysis and Autodock tools (ADT) v1.5.4 and Autodock v4.2 program (Center for Computational Structural Biology, La Jolla, CA, USA). The 3D chemical structure of CDK6 is retrieved from the Protein Data Bank (http://www.pdb.org, accessed on 2 October 2022). The 3D structure of BCP was retrieved from PubChem compound database (http://www.ncbi.nlm.nih.gov/pccompound, accessed on 4 October 2022). The active sites of the target protein were identified using and the obtained results were scored and analyzed based on the predicted binding energy.

### 3.5. RNA Isolation and Quantitative Real-Time PCR

TRIzol Reagent (Invitrogen, Waltham, MA, USA) was used to extract total RNA from the cells. cDNA was synthesized from 500 ng/target of total RNA using a reverse transcriptase (TaKaRa, Maebashi, Japan). RNA was reverse transcribed to cDNA using miScript Reverse Transcription Kit (Qiagen, Hilden, Germany). RNA expression was measured by quantitative real-time PCR in VII 7A applied biosystems (Applied Biosystems, Waltham, MA, USA) using the gene-specific primers (Table 2) and SYBR-Green method (Takara, Japan) according to the manufacturer’s instructions.

### 3.6. Western Blot Analysis

The treated cells were harvested for 24 h and then washed with PBS. Cells were lysed with RIPA lysis buffer and 1× protease inhibitor cocktail, and then the lysate was centrifuged at 8000 rpm for 15 min to remove debris. The lysate supernatant was preserved at −80 °C. Protein concentrations were estimated using Bradford assay. The equivalent of 50 µg of protein extract was separated by SDS-PAGE and then transferred to polyvinylidene difluoride (PVDF) membranes (pore size: 0.45 µm, Bio-Rad, Hercules, CA, USA), which were treated with blocking buffer (5% non-fat dry milk) for 1 h at room temperature before being probed with the appropriate primary antibodies 1:1000 overnight at 4 °C, according to the manufacturer’s protocol. A list of used antibodies is shown in Table 3. The membranes were then washed with TBST buffer and incubated with HRP-conjugated secondary anti-mouse/rabbit IgG antibodies (1:2000 Santa Cruz, Santa Cruz, CA, USA) for 1 h at room temperature. The blots were detected by an enhanced ECL chemiluminescence system (LICOR detection system) and quantified by densitometry using Image J software. The used primary antibodies ZEB-2 mouse monoclonal antibody (1:750, Invitrogen, Waltham, MA, USA), E-Cad rabbit polyclonal antibody (1:1500, Invitrogen, Waltham, MA, USA), β-Catenin rabbit polyclonal antibody (1:1000, Biorbyt, Cambridge, UK), CD-44 rabbit polyclonal antibody (1:500, Invitrogen, Waltham, MA, USA), c-Myc mouse monoclonal antibody (1:1000, Invitrogen, Waltham, MA, USA), SOX-2 mouse monoclonal antibody (1:1000 Invitrogen, Waltham, MA, USA) and β-actin rabbit polyclonal antibody (1:2000, Cell Signaling Technology, Beverly, MA, USA).

### 3.7. Statistical Analysis

Data are expressed as mean ± SD. The significant difference between the DMSO-treated cells and BCP/CDDP-treated cells was analyzed by the two ways analyses of variance. The value of *p* < 0.05 and *p* < 0.01 were considered to be statistically significant and are represented by * and **, respectively

## 4. Discussion

In the present study, BCP was found to be a nontoxic drug to the normal lung cell line, at doses below 100 uM. In the meantime, it can reduce cancer progression in lung cancer cell lines, A549, at doses above 75 µM. However, although it is not a potent tumor suppressor, BCP managed to improve CDDP chemotherapeutic function through regulating the cell cycle, the apoptosis and EMT signaling molecules when being combined with CDDP. In addition, the molecular docking results indicated that BCP is able to interact virtually with the protein kinase, CDK6, that can activate cell cycle and then proliferation activity. Thus, BCP seems to be a potential candidate to work synergistically with CDDP to treat lung cancer.

Several recent studies reported that combining CDDP with other drugs or natural products is a beneficial therapeutic strategy helps to overcome the CDDP side effects and drug resistance [16,17,18]. Therefore, combination of CDDP with other drugs is an approach to overcome drug resistance and reduce toxicity. The combination therapy also results in increased sensitivity of CDDP towards cancer cells. Under the in vivo condition, the combination therapy of CDDP with cilastatin enabled an increase in the dose of CDDP, enhancing its anti-tumor effect by suppressing nephrotoxicity [14]. In addition, treating CDDP-treated A549 cells with the FDA-approved drug, Gleevec, resulted in a synergistic cell killing effect, suggesting that it can potentiate the effect of CDDP on A549 cells [19]. Consistent with that, our data clarified that the combined therapy of BCP and CDDP enabled to reduce the toxic effect of CDDP in normal cells and decreased the migration and invasiveness properties of lung cancer cells. Similarly, the BSE plant extract including α-Guaiene, (−)-guaiol and β-Caryophyllene was reported to responsible for most of the cytotoxic anti-migration and anti-invasion effects of BSE on two lung cancer cell lines [6].

Our results showed that at the gene and the protein expression level, the inhibitor of cell cycle, CDKN1A, is activated by combined treatment of CDDP (12 µM) and BCP (25 and 50 µM, Figure 5) relative to the CDDP-treated cells. In agreement with that, through targeting p21 and cyclin D1, curcumin increased the sensitivity to CDDP and inhibited metastasis and cancer progression in lung cancer cell lines, A549 and H2170 [20]. In addition, we have shown that the combined treatment of CDDP and BCP downregulated the protein level of the apoptosis regulator, BCL-2, and upregulated that of the inhibitor of the apoptosis, BCL-xl. In line with that, the natural hormone, melatonin, enhanced the CDDP-induced cytotoxicity and apoptosis in lung cancer cells, as demonstrated by an increase in S-phase arrest [21].

Change or loss of mitochondrial transmembrane potential is an important step in intrinsic apoptosis. Rho 123 is a fluorescent dye that has a high binding affinity to metabolically active mitochondria. In here, the combination treatment actively controls the membrane damage and increased the release of mitochondrial content in A549 cell lines. In parallel with that, the membrane integrity tends to be lost and the number of intact membranes decreased significantly in combination treatment. In this regard, BCP appears to enhance the intrinsic apoptosis in lung cancer cell lines when being combined with CDDP. However, although this may not agree with our finding, necrosis but not apoptosis was found to be the major cell death in lung cancer cell lines, H661 and A549, stimulated by BS plant extract using Annexin V-FITC/PI detection kit and flow cytometry [6].

On the other hand, the cyclin-dependent kinases are enzymes that are regulated by cyclins and control the cell cycle checkpoints and transitions. The cell cycle inhibitor, CDKN1A, was reported to have a vital role in cell differentiation, apoptosis and DNA repair through regulating cell cycle and it can induce cell cycle arrest in the G1/S or G2/M transitions [22]. The inhibition of cell cycle progression was found to be associated with a higher expression in the levels of CDKN1A expression in lung cancer cell line [23]. In agreement with that, the current study results demonstrate that the combined treatment significantly upregulated the level of CDKN1A, and then, BCP is probably improving the capability of CDDP to control cell cycle progression via upregulating CDKN1A.

The synergism between BCP with CDDP in the present study can also be seen as a significant increase in the level of the epithelial cell marker E-cad that was reduced in CDDP-treated cells and thus BCP appears to prohibit the EMT process that is associated with CDDP chemotherapy. In agreement with that, the combined treatment significantly reduced the level transcription factor, ZEB, elevated after CDDP treatment. ZEB-2 is a promotor of cancer progression and EMT and then combined treatment managed to modulate the effect of CDDP on the mesenchymal transcription factor ZEB-2. These results point out that BCP could improve the cytotoxic effect of CDDP and then solve a problem associated with CDDP treatment, since it is toxic at high doses to both cancer cells and normal cells.

## 5. Conclusions

The synergism between Beta-Caryophyllene and CDDP was achieved via reducing the effective anti-cancer dose of CDDP and improving its chemotherapeutic function through regulating the cell cycle, the apoptosis and EMT signaling molecules. We are currently investigating the action of CDDP and BCP on cyclin-dependent kinase CDK6 and other involved signaling regulatory mechanisms.

## Figures and Tables

**Table 1:** BCP (CID: 5281515) in silico interaction with human cyclin-dependent kinase-6 (inhibitor of CDKN1A).

Protein Receptor	Ligand	Grid Box	Binding Affinity	Interaction Residues	Bond Distance	Interaction Category	Pi Sigma	Alkyl
CDK6 (4EZ5)	Beta-Caryophyllene (CID-No- 5281515)	52.517	−7.8	VAL 27 PHE 98 ILE 19 LEU152 ALA162	4.94 3.88 5.45 5.13 4.09	Hydrophobic Hydrophobic Hydrophobic Hydrophobic Hydrophobic	PHE 98	VAL 27 ILE 19 LEU 152 ALA 162

**Table 2:** List of used primers.

Primer Name	Forward Primer	Reverse Primer	PCR Product Size
CDKN1A	AGGTGGACCTGGAGACTCTCAG	AGGTGGACCTGGAGACTCTCAG	188
E-Cad	GCCTCCTGAAAAGAGAGTGGAAG	TGGCAGTGTCTCTCCAAATCCG	189
ZEB2	AATGCACAGAGTGTGGCAAGGC	CTGCTGATGTGCGAACTGTAGG	231
BCL-2	GATTGTGGCCTTCTTTGAG	CAAACTGAGCAGAGTCTTC	212
BCL-xl	CAGAGCTTTGAACAGGTAG	GCTCTCGGGTGCTGTATTG	167
GAPDH	GTCTCCTCTGACTTCAACAGCG	ACCACCCTGTTGCTGTAGCCAA	195

**Table 3:** List of used antibodies.

Protein Target	Primary Antibody Dilution	Secondary Antibody Dilution	Company
CDKN1A	Rabbit polyclonal antibody (1:500)	HRP conjugated rabbit IgG antibodies	Biorbyt, Cambridge, UK
ECAD	Mouse polyclonal antibody (1:1000)	HRP conjugated Mouse IgG antibodies	Biorbyt, Cambridge, UK
ZEB-2	Mouse monoclonal antibody (1:750)	HRP conjugated mouse IgG antibodies	Biorbyt, Cambridge, UK
BCL2	Rabbit polyclonal (1:1200)	HRP conjugated Rabbit IgG antibodies	Invitrogen, Waltham, MA, USA
BCLxl	Rabbit polyclonal (1:1500)	HRP conjugated rabbit IgG antibodies	Invitrogen, Waltham, MA, USA
Actin	Rabbit polyclonal antibody (1:1000)	HRP conjugated rabbit IgG antibodies	

## Supplementary Materials

The following supporting information can be downloaded at: https://www.mdpi.com/article/10.3390/molecules27238354/s1, Figure S1: Cell lines images.
