# Supplementary figures and images for: Beta-Caryophyllene Enhances the Anti-Tumor Activity of Cisplatin in Lung Cancer Cell Lines through Regulating Cell Cycle and Apoptosis Signaling Molecules
Source: Molecules. 2022 Nov 30;27(23):8354. doi: 10.3390/molecules27238354 (PMC9735510; doi:10.3390/molecules27238354)

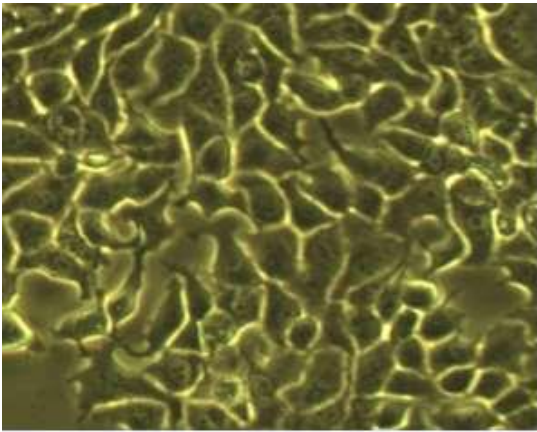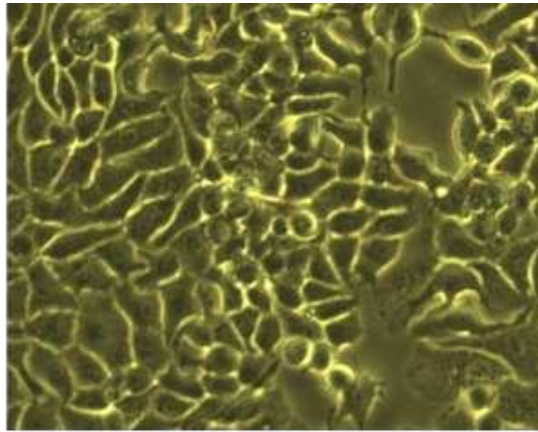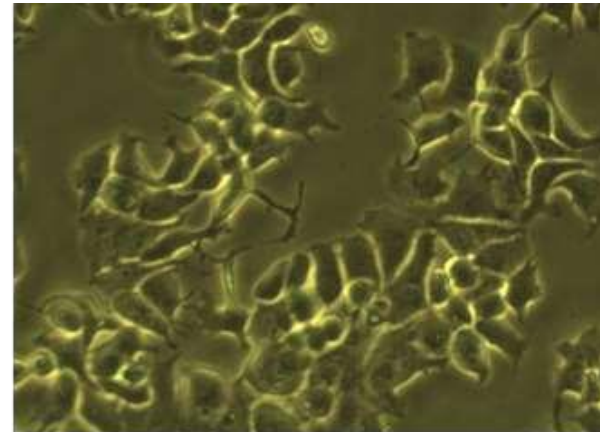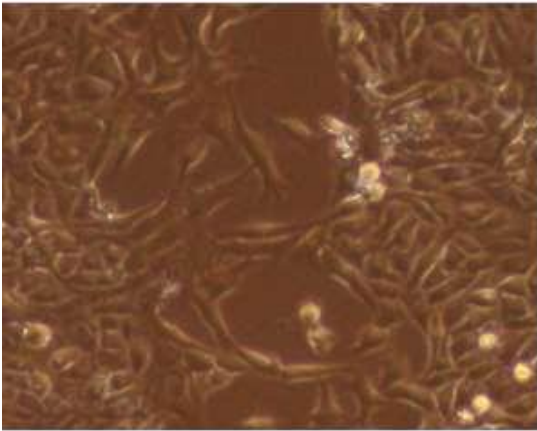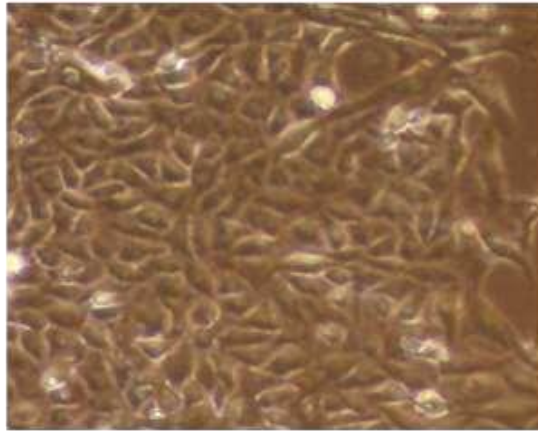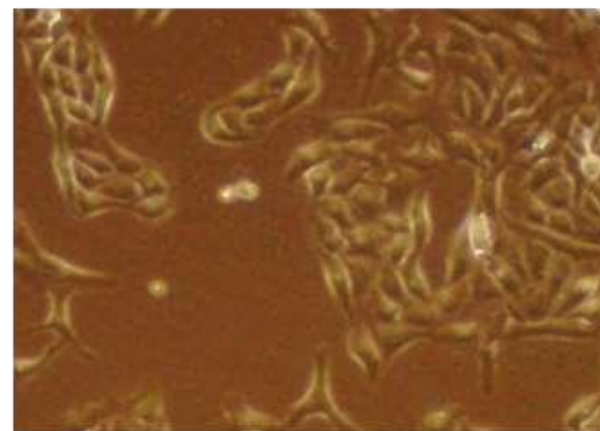

Images for A549 and Beas

**Figure S1.** Cell lines images.

Supplement: Supplementary file 1 [file molecules-27-08354-s001.zip › molecules-2017870-supplementary.pdf]
